# Faster Region-Based Convolutional Neural Network in the Classification of Different Parkinsonism Patterns of the Striatum on Maximum Intensity Projection Images of [^18^F]FP-CIT Positron Emission Tomography

**DOI:** 10.3390/diagnostics11091557

**Published:** 2021-08-28

**Authors:** Byung Wook Choi, Sungmin Kang, Hae Won Kim, Oh Dae Kwon, Huy Duc Vu, Sung Won Youn

**Affiliations:** 1 Department of Nuclear Medicine, Daegu Catholic University Medical Center, Daegu Catholic University School of Medicine, Daegu 42472, Korea; nmchoibw@cu.ac.kr (B.W.C.); kufa77@cu.ac.kr (S.K.); 2 Department of Nuclear Medicine, Keimyung University Dongsan Hospital, Keimyung University School of Medicine, Daegu 42601, Korea; hwkim.nm@gmail.com; 3 Department of Neurology, Daegu Catholic University Medical Center, Daegu Catholic University School of Medicine, Daegu 42472, Korea; dolbaeke@cu.ac.kr; 4 Department of Radiology, Daegu Catholic University Medical Center, Daegu Catholic University School of Medicine, Daegu 42472, Korea; bluecyclone@hanmail.net

**Keywords:** artificial intelligence, dopamine transporter, deep learning, Parkinson’s disease, positron emission tomography

## Abstract

The aim of this study was to compare the performance of a deep-learning convolutional neural network (Faster R-CNN) model to detect imaging findings suggestive of idiopathic Parkinson’s disease (PD) based on [^18^F]FP-CIT PET maximum intensity projection (MIP) images versus that of nuclear medicine (NM) physicians. The anteroposterior MIP images of the [^18^F]FP-CIT PET scan of 527 patients were classified as having PD (139 images) or non-PD (388 images) patterns according to the final diagnosis. Non-PD patterns were classified as overall-normal (ONL, 365 images) and vascular parkinsonism with definite defects or prominently decreased dopamine transporter binding (dVP, 23 images) patterns. Faster R-CNN was trained on 120 PD, 320 ONL, and 16 dVP pattern images and tested on the 19 PD, 45 ONL, and seven dVP patterns images. The performance of the Faster R-CNN and three NM physicians was assessed using receiver operating characteristics curve analysis. The difference in performance was assessed using Cochran’s Q test, and the inter-rater reliability was calculated. Faster R-CNN showed high accuracy in differentiating PD from non-PD patterns and also from dVP patterns, with results comparable to those of NM physicians. There were no significant differences in the area under the curve and performance. The inter-rater reliability among Faster R-CNN and NM physicians showed substantial to almost perfect agreement. The deep-learning model accurately differentiated PD from non-PD patterns on MIP images of [^18^F]FP-CIT PET, and its performance was comparable to that of NM physicians.

## 1. Introduction

Parkinsonism is an umbrella term for a symptom complex that includes tremor at rest, bradykinesia, rigidity, and postural instability [1]. Although the underlying causes of parkinsonism are diverse, idiopathic Parkinson’s disease (PD) is by far the most common cause, followed by atypical parkinsonism (APD). The differential diagnosis of parkinsonism further includes essential tremor, vascular parkinsonism (VP), drug-induced parkinsonism, and other disorders [2]. Despite recent advances in neuroimaging and genetic analysis, this differential diagnosis remains primarily based on clinical assessment. All the mentioned conditions show a considerable overlap of their clinical features in the early stage, leading to frequent changes in the diagnosis of patients with parkinsonism during the first years [3,4]. The correct differentiation of PD from other parkinsonism causes is not only essential in the therapy and prognosis of patients but is also the foundation of effective clinical, pharmacological, and epidemiological research.

The definitive diagnosis of PD relies on histological examination of brain tissue, which can only be performed post-mortem. Therefore, neuroimaging techniques are increasingly used in the workup of PD and can substantially support the clinical diagnosis [2]. Various neuroimaging modalities, such as magnetic resonance imaging (MRI) of the brain, single-photon emission computed tomography (SPECT), and positron emission tomography (PET), improve the accuracy of the diagnosis in patients with parkinsonism [5,6]. Several radiopharmaceuticals, which target the presynaptic dopamine transporter (DAT) mediating the reuptake of dopamine from the synaptic cleft, derived from tropane and cocaine analogs, including [^123^I]N-ω-fluoropropyl-2β-carbomethoxy-3β-(4-iodophenyl)nortropane ([^123^I]fluoropropylcarbomethoxyiodophenylnortropane (FP-CIT), [^123^I]carbomethoxyiodo-phenyl-tropane ([^123^I]β-CIT), and [^18^F]FP-CIT have been used to assess the DAT location and density in the striatum, and their binding to DATs in the striatum is an important clue in the differential diagnosis of parkinsonism [7,8,9]. The DAT distribution density in the striatum can be evaluated on SPECT and PET scans quantitatively with the specific striatal binding ratio and visually considering the characteristic striatum shape features [8,9]. DAT-SPECT scans show different DAT patterns in the conditions described and may contribute to the differentiation between PD and APD [10]. On maximum-intensity projection (MIP) images of [^18^F]FP-CIT PET scans, characteristic shape features (“rabbit” sign) may be used as diagnostic clues to differentiate PD from APD [9]. Therefore, the visual assessment based on characteristic shape features on DAT-SPECT and PET scans may assist in differentiating PD from other causes of parkinsonism.

Recently developed deep-learning methods may assist the computer-aided diagnosis of medical images [11]. The faster region-based convolutional neural network (Faster R-CNN) is an object detection algorithm based on deep learning and shows superior performance over previous versions (R-CNN and Fast CNN) by using region proposal networks (RPNs) [12]. Several studies using the Faster R-CNN method have demonstrated its excellent performance across a range of visual tasks in radiology [13,14]. Considering the object classification ability of Faster R-CNN that discriminates certain features in the input images, we hypothesized that Faster R-CNN might be highly effective in distinguishing PD from other parkinsonian syndromes on DAT-SPECT and PET scans. Although DAT-SPECT performed well in the visual assessment and diagnosis of PD, DAT-PET may more efficiently assist in the visual assessment using Faster R-CNN because the spatial resolution of a PET scan is superior to that of SPECT [15]. 

MIP images of SPECT and PET scans are commonly used to identify the shape features of radiopharmaceuticals in the target organs, including that of striatal DAT binding [9]. Furthermore, MIP images are generally composed of only a few dozen images, and their use may be more efficient in Faster R-CNN analysis than transverse or other plane images, as their number of these plane images is usually greater than that of MIP images. However, studies using MIP images of DAT-PET scans in Faster R-CNN analysis of the striatum in patients with parkinsonism are lacking. 

Therefore, this study aimed to evaluate the performance of Faster R-CNN in differentiating PD patterns from other parkinsonism patterns based on MIP images of [^18^F]FP-CIT PET scans and compare it with the visual assessment results by three nuclear medicine (NM) physicians.

## 2. Materials and Methods

### 2.1. Subjects

Six hundred and sixty-one consecutive patients who visited the movement disorder clinic of our hospital and underwent [^18^F]FP-CIT PET for the evaluation of parkinsonism symptoms between December 2016 and August 2019 were considered.

**Figure 1:**
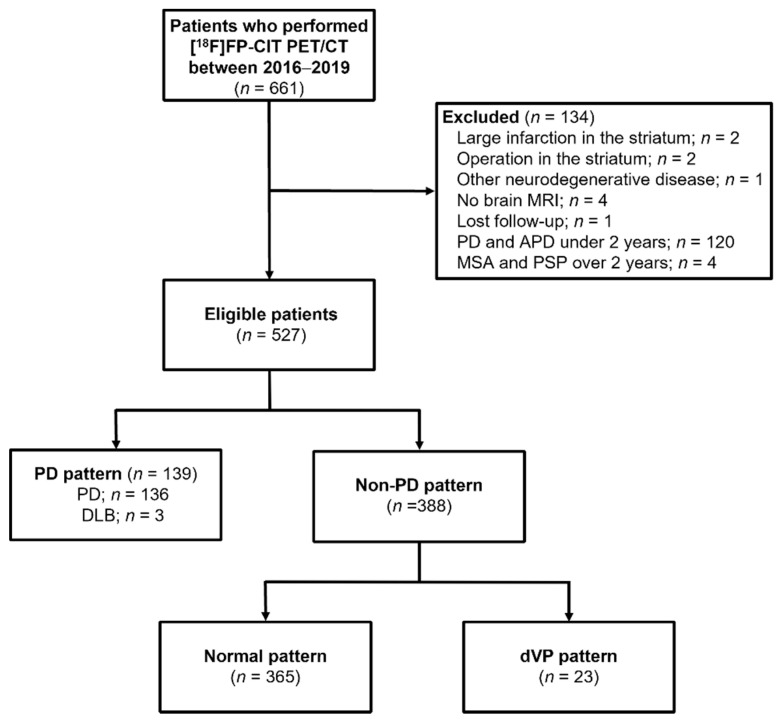
Flow chart of patients in this retrospective study on the use of a faster region-based convolutional neural network model in the classification of patients with parkinsonism (*n* = 661).

The exclusion criteria were as follows: (1) diagnosis of PD or dementia with Lewy bodies (DLB) within two years of onset; (2) multiple system atrophy, progressive supranuclear palsy, or corticobasal degeneration; (3) no available brain MRI within three months before or after the [^18^F]FP-CIT PET scan; (4) significant structural change in the striatum after surgery, infection, or large cerebral infarction or intracranial hemorrhage; (5) insufficient data or records. All subjects had been assessed by a neurologist (O.D.K.) specialized in movement disorders, and the diagnoses of PD, DLB, and secondary parkinsonism were based on current diagnostic criteria and the patients’ clinical presentation [16,17,18,19]. Based on these criteria, we excluded 134 patients and included 527 eligible patients in the analysis (Figure 1).

We obtained the medical records of all included subjects from the electronic healthcare information system and extracted the following data: sex, age, follow-up periods, and severity measured according to the Hoehn and Yahr (H&Y) scale when performing [^18^F]FP-CIT PET. The institutional review board approved this study and waived the need to obtain written informed subject consent due to its retrospective design.

### 2.2. [^18^F]FP-CIT Positron Emission Tomography/Computed Tomography Acquisition

As part of our clinical routine, all patients had undergone [^18^F]FP-CIT PET/computed tomography (CT) scans using an integrated PET/CT system (Discovery IQ; GE Healthcare, Chicago, IL, USA) and stopped their antiparkinsonian drugs 12 h before the examination. Image acquisition was started immediately (early phase) and 3 h (late phase) after the intravenous injection of [^18^F]FP-CIT (185 MBq). Emission PET data were acquired in the three-dimensional mode for 10 min after brain CT, which was performed in the spiral mode at 120 kVp and 60 mA using the ASiR program for attenuation correction. [^18^F]FP-CIT PET images were reconstructed using a Bayesian penalized likelihood image reconstruction algorithm (Q. Clear, GE Healthcare, Chicago, IL, USA) with a 256 × 256 matrix.

### 2.3. Image Classification and Data Annotation

**Figure 2:**
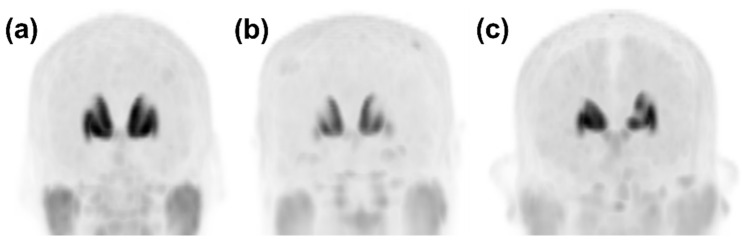
Representative anteroposterior maximum-intensity projection image patterns from [^18^F]FP-CIT PET. (**a**) In the case that there was no focal defect or a decrease in dopamine transporter (DAT) binding of the striata, the pattern was classified as overall normal. (**b**) A PD pattern was defined as the typical DAT binding loss in the dorsal posterior putamen with relative sparing of a DAT binding loss in the ventral putamen. (**c**) A dVP pattern was defined as any focal DAT binding defect or decrease in the striatum that differed from the typical PD pattern. DAT, dopamine transporter; PD, idiopathic Parkinson’s disease; dVP, vascular parkinsonism with prominent defect or decreased dopamine transporter binding mimicking PD.

As part of this study on the training and testing of Faster R-CNN, all images taken during the late phase after intravenous injection of [^18^F]FP-CIT were classified and pre-processed as follows: The [^18^F]FP-CIT PET images were classified into PD and non-PD patterns according to patients’ diagnosis [9,20]. Images of patients with DLB were classified as a PD pattern [20]. Among the non-PD patterns, the images that met the following criteria were further classified as a VP pattern with definite defects or prominently decreased DAT binding (definite VP/dVP) by an experienced NM physician (B.W.C.): (1) patients had been diagnosed with VP; (2) “punched out” or segmentally decreased DAT binding of [^18^F]FP-CIT in the striatum but not matched to a PD pattern; (3) corresponding high signal intensities in the T2-weighted brain MRI suggesting cerebral infarction. The images showing a non-PD pattern, except for the dVP pattern, were classified as overall normal (ONL). The typical image patterns are presented in Figure 2. 

After classification of the pattern of images, one anteroposterior MIP image of the [^18^F]FP-CIT PET scan of all patients was extracted from the picture archiving and communication system and saved as a JPEG file. We then created training and test sets of PD, ONL, and dVP patterns that were serially collected for each pattern according to the date of the scan. Further, an NM physician (B.W.C.) imported the image data into the web-based VGG Image Annotator (VIA) tool [21], identified the ventral and dorsal striata in the anteroposterior MIP image, and manually drew minimum rectangle regions of interests (ROIs) around each striatum. After completing the localization of the rectangle ROIs, a raw comma-separated values (CSV) file containing bounding box coordinates (x, y, width, and height) was created. The bounding box coordinates in the raw CSV file were converted into the required format (x, y, x + width, and y + height) with the pattern label identified for each image, to be read by the Python-based Faster R-CNN pipeline. 

### 2.4. Faster Region-Based Convolutional Neural Network (Faster R-CNN) Architecture Construction

**Figure 3:**
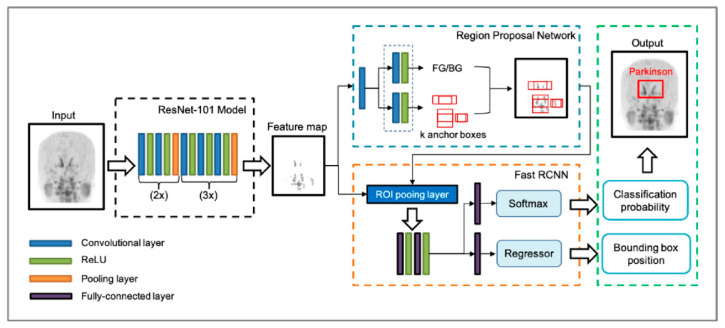
The architecture of the faster region-based convolutional neural network (Faster R-CNN) used. The Faster R-CNN consists of the region-proposal network (RPN) and the fast R-CNN. From the input image, a ResNet-101 model without fully connected layers was utilized to extract a feature map. RPN learns to determine whether an object is present in the input image by placing a set of anchors. As the network propagates each pixel in the feature map, these anchors are checked to determine the objectness score and refine the anchor’s coordinates of rectangles in the region of interest (ROI). The output features from the ROI pooling layer are fed into the fully connected layers with the softmax and regressor branches, finally generating classification probability and bounding box position. ReLU, rectified linear unit.

The Faster R-CNN consisted of the RPN and the Fast R-CNN (Figure 3). Using the input images, the RPN extracted the feature map that was fed into the backbone convolutional neural network. The ResNet-101 model was utilized in the Faster R-CNN to extract features from the MIP image of the [^18^F]FP-CIT PET. The RPN learns every point in the output feature map to determine whether an object is present on the input image at the corresponding location by placing a set of anchors on the input image for each location on the feature map. As the network propagates each pixel in the feature map, these anchors are checked to determine the objectness score to refine the anchor’s coordinates of the bounding boxes as the ROI. The Fast R-CNN detector also consists of a CNN backbone, an ROI pooling layer, and fully connected layers followed by two branches for classification probability and bounding box regression. The bounding box proposals from the RPN are used to pool features from the backbone feature map implemented by the ROI pooling layer. The ROI pooling layer works by taking the region corresponding to a proposal from the backbone feature map, dividing this region into a fixed number of sub-windows, and performing max pooling over these sub-windows. Finally, the output features from the ROI pooling layer are fed into the fully connected layers and the softmax and bounding box branches. 

### 2.5. Training, Validation, and Testing of Faster R-CNN

**Figure 4:**
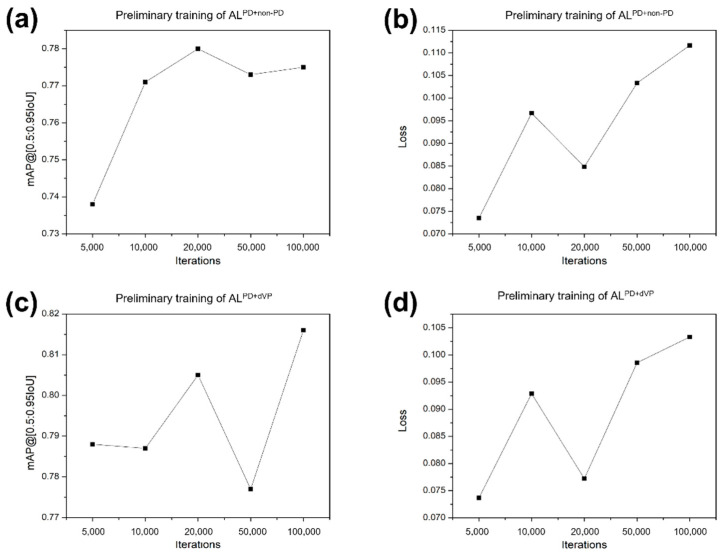
Mean average precision values and loss function values of the preliminary training. (**a**,**b**) Algorithm (AL)^PD+non-PD^ and (**c**,**d**) AL^PD+dVP^ at various iterations (5000, 10,000, 20,000, 50,000, and 100,000). AL^PD+non-PD^ and AL^PD+dVP^ represent the algorithm classifying parkinsonism in the training sets composed of the two groups Parkinson’s disease (PD) + non-PD and PD + definite vascular parkinsonism with prominent defect or decreased dopamine transporter binding mimicking PD (dVP). mAP, mean average precision.

Deep learning was performed in the following environment using the following system: A central processing unit Intel^®^ Core™ i7-8700K, 12 cores 3.70 GHz; graphics processing unit Geforce RTX™ 2080 Ti 12 GB (NVIDIA^®^, Santa Clara, CA, USA), Ubuntu 18.04 operating system (Canonical Ltd., London, UK), CUDA 10.1 computing environment (NVIDIA^®^, Santa Clara, CA, USA), TensorFlow 1.12, and Python 3.6. After the image classification and data annotation, the datasets were divided into training/validation and test sets, and the training/validation set was further divided into training and validation subsets for the 4-fold cross-validation (the ratio of the training to validation subsets was 3:1). Three groups of PD, ONL, and dVP patterns were randomly partitioned into four equal-sized independent subsets. For training and validation, each pattern was used in a training algorithm for the PD versus non-PD classification (AL^PD^^+^^non-PD^) and the PD versus dVP classification (AL^PD^^+^^dVP^), respectively. Each model was trained with varied iterations of 5000, 10,000, 20,000, and 50,000 steps. This process was performed four times until each data proportion in the entire dataset had been used for validation once. The validation results were then presented with mean average precision (mAP) values and the loss function at each training session in each algorithm. The variations in the mAP at the intersection over the union in the range of 50–95% and the change trend in the loss function values at varied iterations (5000 to 100,000) were calculated (Figure 4). 

In the training, high mAPs and low loss function values were recorded at 20,000 iterations for the AL^PD+non-PD^ (0.780 and 0.085, respectively) and AL^PD+dVP^ (0.805 and 0.077, respectively). In the validation session, the iteration of 20,000 was chosen with optimal mAP and loss function values (Table 1). On comparing the loss function values in the AL^PD-non-PD^, there was a difference between training and validation (*p* < 0.05), while no significant difference was found for the AL^PD+dVP^.

With the test set, AL^PD+non-PD^ and AL^PD+dVP^ produced a probability range 0–1 for either PD or non-PD and PD or dVP, respectively. Each pattern score of PD and non-PD in AL^PD+non-PD^ was calculated as follows:

(1) PD probability⁄(non-PD probability+PD probability)

and

(2) Non-PD probability⁄(non-PD probability+PD probability)

Each pattern score of PD and dVP in AL^PD+dVP^ was calculated as follows:

(3) PD probability ⁄(PD probability+dVP probability)

and

(4) dVP probability ⁄(PD probability+dVP probability)

### 2.6. Pattern Classification by the NM Physicians

Concomitant to the classification of the Faster R-CNN, the images were independently evaluated by three board-certificated NM physicians who possessed 15, 15, and 12 years of experience, respectively, in the interpretation of NM images. After waiting for one month following the image pattern classification and lesion masking to reduce recall by BWC, the MIP images of each patient, which were the same as those in the test set evaluated using the Faster R-CNN, were provided to the three physicians, and they were asked to distinguish between PD and non-PD patterns. To avoid interpretation bias, the NM physicians had no access to clinical information, results, or the images created using other modalities, such as brain CT and MRI, but information regarding the classification of the images as having PD or non-PD patterns and further classification of the latter as having ONL and dVP patterns was provided to NM physicians for comparison with the results of Faster R-CNN. After waiting for another month to reduce recall bias, a test set of images for the classification of PD and dVP patterns was provided to the same physicians under the same conditions as those in the previous test, informing them that this test set consisted of only PD and dVP patterns. 

The classification results of each NM physician were recorded and compared with those of the Faster R-CNN.

### 2.7. Statistical Analyses

Numeric data are expressed as the mean ± standard deviation (SD) and were compared using the independent samples *t*-test. Sex differences were compared between patterns using Fisher’s exact test. Receiver operating characteristic (ROC) curve analysis was used to assess the diagnostic performance of the Faster R-CNN and NM physicians, and the area under the curve (AUC) with the standard error was calculated. The DeLong method with the Bonferroni correction was applied in the pairwise comparison of the ROC curves between Faster R-CNN and each NM physician. Cochran’s Q test was performed to assess the differences in diagnostic performance between the Faster R-CNN and the physicians. Fleiss’ κ coefficient was used to evaluate the inter-rater reliability between the Faster R-CNN and all NM physicians. A *p*-value < 0.05 indicated statistically significant. All statistical analyses were performed using the IBM Statistical Package for the Social Sciences for Windows, version 26.0 (IBM Corp., Armonk, NY, USA).

## 3. Results

### 3.1. Patients’ Clinical Characteristics

Among the 527 patients included, 136 were diagnosed with PD, three with DLB, and 388 with essential tremor and secondary parkinsonism. Based on the clinical diagnosis, MIP images of the [^18^F]FP-CIT PET of 139 subjects were classified as having PD patterns and 388 as having non-PD patterns (Figure 1). The clinical characteristics of the subjects according to PD and non-PD patterns are summarized (Table 2). The non-PD patterns were further classified as 365 ONL and 23 dVP patterns. Among them, 120 PD, 320 ONL, and 16 dVP pattern images were used in the training/validation set and 19 PD, 45 ONL, and 7 dVP pattern images were used in the test set. 

### 3.2. Calculated Pattern Score of Each Image Using the Faster R-CNN

In the AL^PD+non-PD^, PD patterns had a PD score of 0.932 ± 0.188 and a non-PD score of 0.068 ± 0.188. The non-PD patterns had a PD score of 0.007 ± 0.017 and a non-PD score of 0.993 ± 0.017. There was a misclassification in one PD pattern, in which the PD and non-PD scores were 0.239 and 0.761, respectively. In the AL^PD-dVP^, PD patterns had a PD score of 0.953 ± 0.123 and dVP score of 0.047 ± 0.123. The dVP patterns had a dVP score of 0.562 ± 0.366 and a PD score of 0.438 ± 0.366. Two dVP patterns showed a PD score of 0.903 and 0.991, respectively, and a dVP score of 0.097 and 0.009, respectively. Except for these two dVP patterns, the other dVP patterns had a dVP score of 0.766 ± 0.136 and PD score of 0.234 ± 0.136. 

PD and non-PD scores of AL^PD+non-PD^ and PD and dVP scores of AL^PD-dVP^ were selected for the classification of PD and non-PD and PD and dVP, respectively. A pattern score ≥0.5 was regarded as a positive classification for each pattern, and a score <0.5 was regarded as negative. 

### 3.3. Performance Comparison between the Faster R-CNN and NM Physicians

**Figure 5:**
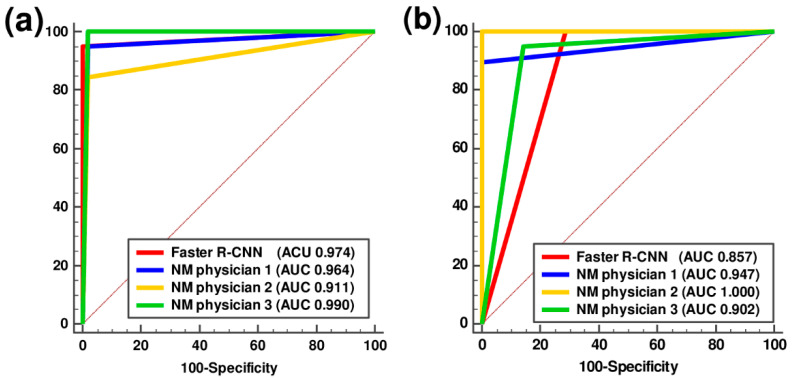
Receiver-operating characteristic curve analysis of the faster region-based convolutional neural network and three nuclear medicine physicians in classifying parkinsonism. (**a**) Parkinson’s disease (PD) and non-PD and (**b**) PD and definite vascular parkinsonism with prominent defect or decreased dopamine transporter binding mimicking PD in the test session. AUC, area under the curve; Faster R-CNN, faster region-based convolutional neural network; NM, nuclear medicine.

The Faster R-CNN achieved 94.7% sensitivity, 100% specificity, and 98.6% accuracy in classifying PD and non-PD patterns (Table 3). In the classification of PD and dVP patterns, the Faster R-CNN showed 100.0% sensitivity, 71.4% specificity, and 92.3% accuracy. The sensitivity, specificity, and accuracy of each of the three NM physicians in classifying the different patterns are shown in Table 3. The ROC curve analysis for the Faster R-CNN and the three NM physicians in their classification of the different patterns is shown in Figure 5. 

The pairwise comparisons of the AUCs for each physician with those of the Faster R-CNN showed no statistically significant differences (Table 4). The rates of disagreement in distinguishing PD from non-PD patterns and PD from dVP patterns between the Faster R-CNN and NM physicians were 9.9% and 19.2%, respectively. The Cochran’s Q test showed no significant differences between the Faster R-CNN and NM physicians for classification of both PD and non-PD image patterns and PD and dVP image patterns (*p* = 0.436 and 0.311, respectively). 

The analysis of the overall inter-rater reliability between the Faster R-CNN and NM physicians showed almost perfect agreements in distinguishing PD from non-PD patterns (Fleiss κ coefficient, 0.866 (*p* < 0.001)) and substantial agreement in distinguishing PD from dVP patterns (Fleiss κ coefficient, 0.739 (*p* < 0.001)). 

## 4. Discussion

To the best of our knowledge, this is the first time that deep learning was applied to [^18^F]FP-CIT PET images in the differential diagnosis of parkinsonism. We evaluated the performance of the Faster R-CNN in distinguishing PD from other patterns in parkinsonism using one anteroposterior MIP image of each patient’s [^18^F]FP-CIT PET. The algorithm’s performance was comparable to that of three experienced NM physicians and confirmed that Faster R-CNN effectively distinguishes PD from other parkinsonian syndromes on PET scans. We conclude that the Faster R-CNN classification of one anteroposterior MIP image of the [^18^F]FP-CIT PET may assist NM physicians in accurately interpreting DAT images.

Studies using DAT-SPECT scans and various computational techniques have shown excellent performance with high accuracy in the classification of parkinsonism patterns [22,23,24,25,26]. The accuracy of the Faster R-CNN in distinguishing the PD pattern from other patterns was comparable to previous findings. Furthermore, although the specificity and accuracy in differentiating PD from dVP patterns were slightly lower than that of the other model in our study, the Faster R-CNN classifications were comparable to those of NM physicians. 

PD patients show characteristic shape features with decreased DAT binding in the posterior putamen and relatively preserved DAT binding in the ventral putamen on DAT PET scans [9]. However, the lesions resulting from cerebral infarction in the striatum are usually of irregular shape, and it may be difficult to identify characteristic shapes, especially in the small population of our study. Object detection frameworks based on deep learning, such as Faster R-CNN, require a large number of images for optimal performance because they use multiple training models to find rules and identify characteristic shape features [12,13]. Therefore, further studies with large numbers of images with a dVP pattern may improve the classification performance of Faster R-CNN. 

We trained the binary classification models by pairing two patterns (PD and non-PD and PD and dVP) from the training dataset libraries simultaneously. When evaluating the performance of the binary classification models in the test session, these models were exposed to the images that were not trained in the training session. However, these new exposures were overcome by introducing a pattern score calculated from the probability of binary classification models and comparing the pattern score to the actual classification of each pattern. We confirmed that the classification using the pattern score was effective, and it may be applied to classification problems in other studies using medical images. Although there were a few misclassifications based on the pattern score, the accuracy of the classification might improve by increasing the sample size of the training datasets and using study populations with more equally distributed patterns.

Inter-rater reliability is a critical aspect of any new classification method because an observer’s interpretation of DAT scans may affect the clinical decision making in these patients [26,27,28]. Excellent inter-rater reliability was found between three independent observers in the binary classification of DAT-SPECT scans as “normal” and “abnormal” [28], while the other studies showed substantial discrepancies and suboptimal results for the inter-rater reliability [26,27]. In contrast, a previous study with DAT PET scans showed good inter-rater reliability in the visual analysis by NM physicians when discriminating PD from progressive supranuclear palsy and multiple system atrophy [9]. In our study, the disagreement rates among the NM physicians ranged from 6.3 to 11.5% and those between the Faster R-CNN and the NM physicians from 7.8 to 19.2%. The inter-rater reliability was comparable or superior to that reported earlier [26,27,28]. Based on the high accuracy and inter-rater reliability, Faster R-CNN may have a clinical impact on PD diagnosis.

In clinical practice, physicians usually interpret DAT-SPECT and PET scan images using both visual and quantitative analyses, and because human observers can visually acquire information similar to that of quantitative image analysis [29]. Therefore, the higher the image resolution, the more information obtained from visual analysis. The spatial resolution of PET images is generally two to three times better than that of SPECT [15]. The better spatial resolution of the DAT PET images enables NM physicians to perform a more sophisticated visual subregion analysis [9]. Similarly, DAT scans with better image resolution than DAT-SPECT may help Faster R-CNN to find more characteristic shape features during training. 

Recent machine-learning studies using quantitative parameters, such as the striatal binding ratio of DAT-SPECT, showed high accuracy in the classification of PD [24,25,30]. The use of quantitative analysis is more objective than relying on visual analysis alone. However, there are some general limitations to the use of quantitative data, because they may be affected by image data processing, imaging equipment, and clinical factors, such as the age and sex of patients [7,31]. Recent studies reported that the visual assessment of shape features provides valuable information in PD diagnosis [10,24]. We could not perform quantitative analysis because information essential for quantitative analysis is lost during the transformation of the images. Nevertheless, Faster R-CNN demonstrated high accuracy without considering the striatal binding ratio.

Anatomic imaging studies using 3T T1-weighted brain MRI showed high performance for the classification of PD patients from control subjects [32,33]. Although these studies used different artificial intelligence algorithms and target regions, the overall accuracy was over 90% and comparable to the results of the present study. These results are extremely encouraging because they suggest that artificial intelligence based on both anatomic and functional images could be helpful in practical clinical situations. Nevertheless, a further study involving both anatomic and functional images is needed to validate this hypothesis. 

Our study has several limitations. First, because the clinical diagnosis of PD patients may change during follow-up, long-term follow-up is preferred in clinical practice. The minimum follow-up of patients with PD patterns in this study was at least two years, but the patients with non-PD patterns had a relatively short follow-up depending on the course of their disease. Second, the NM physicians used only one MIP image, whereas the conventional approach uses both early and late-phase tomography images. The accuracy of the classification by NM physicians might improve when the conventional method is applied, using both visual and quantitative analysis. However, since each NM physician accurately classified patterns in more than 90% of the images, it is not expected that using the conventional methods will make a significant difference. Third, this study was based on a retrospective review and data in a single hospital with a relatively low number of patients with PD and dVP patterns, which may have resulted in selection bias. Finally, a significant difference was observed between patients with PD and non-PD patterns, and no statistical adjustment was performed. Although age is a well-known risk factor for PD and other causes of parkinsonism [19], the classification of the image pattern in this study was performed based on the clinical diagnosis, which considers the patient’s age and other clinical features. Furthermore, a different DAT distribution density in the striatum on SPECT and PET scans was reported based on the disease rather than age [2,9]. Overall, the main purpose of this study was to compare the performance of Faster R-CNN and NM physicians using only image patterns without other clinical data. Therefore, the effect of age is supposedly minimal on the results of the present study.

## 5. Conclusions

In conclusion, we present a novel, easily reproducible, and user-independent deep-learning model using one anteroposterior MIP image of the [^18^F]FP-CIT PET. This model accurately differentiated PD patterns from other patterns in more than 90% of images. Moreover, the classification of the Faster R-CNN showed substantial to almost perfect agreement with that of three NM physicians. Faster R-CNN, an objective automated system with high accuracy, may provide reliable support to clinicians in the diagnostic process in PD patients. This technique can be used in connection with different medical imaging modalities, and its application can be extended to the imaging analysis of various other diseases. A multi-center study with a large patient population and long-term follow-up is needed to validate our findings. 

## Figures and Tables

**Table 1:** Mean average precision and loss function during the training session using the faster region-based convolutional neural network with 4-fold cross-validation.

	Mean Average Precision				Loss of Function			
	AL^PD+non-PD^		AL^PD+dVP^		AL^PD+non-PD^		AL^PD+dVP^	
	Train	Validation	Train	Validation	Train	Validation	Train	Validation
Model 1	0.762	0.768	0.794	0.794	0.108	0.096	0.09	0.088
Model 2	0.743	0.755	0.724	0.754	0.101	0.088	0.124	0.116
Model 3	0.769	0.765	0.782	0.789	0.094	0.089	0.081	0.082
Model 4	0.761	0.784	0.790	0.799	0.107	0.095	0.103	0.102
Mean	0.759	0.768	0.772	0.784	0.103	0.092	0.100	0.097
SD	0.011	0.012	0.032	0.020	0.006	0.004	0.019	0.015
*p*-value	NS		NS		*p* < 0.05		NS	

AL^PD+non-PD^ and AL^PD-dVP^ are trained algorithms used to distinguish idiopathic Parkinson’s disease (PD) from non-PD and PD from definite vascular parkinsonism with prominent defect or decreased dopamine transporter binding mimicking PD, respectively. Models 1, 2, 3, and 4 represent each run of the 4-fold cross-validation. *p* < 0.05 indicates a statistically significant difference. PD, idiopathic Parkinson’s disease; dVP, definite vascular parkinsonism with prominent defect or decreased dopamine transporter binding mimicking PD; Train, training session; SD, standard deviation; NS, not significant.

**Table 2:** Baseline characteristics of patients (*n* = 527) undergoing medical imaging for the differential diagnosis of parkinsonism.

	PD Pattern (*n* = 139)	Non-PD Pattern (*n* = 388)	*p*-Value
Age of onset (years)	65.4 ± 9.1	69.1 ± 11.6	<0.001
Sex, male/female (*n*)	55/84	133/255	0.264
Follow-up duration (months)	56.3 ± 38.0	12.9 ± 10.5	<0.001
Modified Hoehn and Yahr stage	2.2 ± 0.9	NA	

Values are reported as the mean ± standard deviation unless otherwise indicated. PD, idiopathic Parkinson’s disease; NA, not applicable.

**Table 3:** Diagnostic performance of the faster region-based convolutional neural network and nuclear medicine physicians based on image patterns.

	Reader	Sens (%)	Spec (%)	PPV (%)	NPV (%)	Accuracy (%)
PD vs. non-PD	Faster R-CNN	94.7	100.0	100.0	98.1	98.6
	NM Physician 1	94.7	98.1	94.7	98.1	97.2
	NM Physician 2	84.2	98.1	94.1	94.4	94.4
	NM Physician 3	100.0	98.1	95.0	100.0	98.6
PD vs. dVP	Faster R-CNN	100.0	71.4	90.5	100.0	92.3
	NM Physician 1	89.5	100.0	100.0	77.8	92.3
	NM Physician 2	100.0	100.0	100.0	100.0	100.0
	NM Physician 3	94.7	85.7	94.7	85.7	92.3

PD, idiopathic Parkinson’s disease; dVP, definite vascular parkinsonism with prominent defect or decreased dopamine transporter binding mimicking PD; Faster R-CNN, faster region-based convolutional neural network; Sens, sensitivity; Spec, specificity; PPV, positive predictive value; NPV, negative predictive value; NM, nuclear medicine.

**Table 4:** Pairwise comparison of receiver operating characteristics curves of the faster region-based convolutional neural network and nuclear medicine physicians based on image patterns.

		Difference between Areas	SE	95% CI	Z Statistics	*p*-Value *
PD vs. non-PD	NM Physician 1	0.0096	0.0394	−0.0677 to 0.0869	0.244	0.807
	NM Physician 2	0.0622	0.0535	−0.0426 to 0.1670	1.163	0.245
	NM Physician 3	0.0167	0.0280	−0.0382 to 0.0716	0.596	0.551
PD vs. dVP	NM Physician 1	0.0902	0.0991	−0.1040 to 0.2840	0.911	0.362
	NM Physician 2	0.1430	0.0922	−0.0379 to 0.3240	1.549	0.121
	NM Physician 3	0.0451	0.1330	−0.2160 to 0.3060	0.339	0.735

* Comparison with the faster region-based convolutional neural network; ROC, receiver operating characteristic; PD, idiopathic Parkinson’s disease; dVP, definite vascular parkinsonism with prominent defect or decreased dopamine transporter binding mimicking PD; NM, nuclear medicine; SE, standard error; CI, confidence interval.

## Data Availability

The data presented in this study are available on request from the corresponding author.
